# Stationäre Versorgung in der Kinder- und Jugendpsychiatrie – wer hat Platz?

**DOI:** 10.1007/s40211-022-00443-y

**Published:** 2022-11-08

**Authors:** Kathrin Sevecke, Anna Wenter, Isabel Böge

**Affiliations:** 1 Medizinische Universität Innsbruck, Christoph-Probst-Platz, Innrain 52, 6020 Innsbruck, Österreich; 2 Abteilung für Kinder- und Jugendpsychiatrie, Psychotherapie und Psychosomatik, Tirol Kliniken, Milser Straße 10, 6060 Hall i. T., Österreich; 3 Universitätsklinikum Graz, Auenbruggerplatz 1, 8036 Graz, Österreich; 4 Abteilung für Kinder- und Jugendpsychiatrie und Psychotherapie, LKH II, Wagner-Jauregg Platz 1, 8053 Graz, Österreich; 5 Institut für Psychologie, Fakultät für Psychologie und Sportwissenschaft, Leopold-Franzens-Universität Innsbruck, Universitätsstraße 5–7, 6020 Innsbruck, Österreich

**Keywords:** Kinder und Jugendliche, Psychische Gesundheit, Stationäre Versorgung, Bettenmessziffer, Children and adolescents, Mental health, Inpatient care, Bed index

## Abstract

**Hintergrund und Fragestellung:**

Im vorliegenden Artikel wird die stationäre kinder- und jugendpsychiatrische Versorgungslandschaft in Österreich vorgestellt, aktuelle Versorgungsdaten mit Stand Juni 2022 erhoben und gemessen am Bedarf beleuchtet.

**Methode:**

Im Juni 2022 haben die Autor:innen den aktuellen Ist-Stand an den österreichischen kinder- und jugendpsychiatrischen Primariaten schriftlich erhoben. Hierbei wurden sowohl systemisierte Betten und Tagklinikbetten als auch die Anzahl der Fachärzt:innen und Assistenzärzt:innen berücksichtigt.

**Ergebnisse:**

Es wurde eine Bettenmessziffer von 0,05 Betten/1000 Einwohner:innen (Stand 6/2022) berechnet, die unter den vom Österreichischen Strukturplan Gesundheit vorgegebenen Planungsrichtwerten liegt und dem – durch die Pandemie weiter angestiegenen – realen Bedarf nicht entspricht. Darüber hinaus waren im Juni 2022 österreichweit 40 Betten wegen Personalmangels geschlossen und 26,5 Ausbildungsstellen unbesetzt.

**Diskussion und Schlussfolgerungen:**

Auch wenn mit 07.02.2022 der Ausbildungsschlüssel im Rahmen der Mangelfachverordnung befristet bis 31.05.2027 auf 1:2 angehoben wurde, ist eine hinreichende Versorgung des – gerade in den Zeiten der Pandemie gestiegenen – kinder- und jugendpsychiatrischen Bedarfs so kaum zu gewährleisten. Es ist dringend notwendig, dass Maßnahmen zur Verbesserung der kinder- und jugendpsychiatrischen Behandlungsmöglichkeiten ergriffen werden. Um nicht nur Notfallmanagement zu betreiben, sondern eine angemessene Versorgung der zunehmenden Zahl an manifest psychisch erkrankten Kindern und Jugendlichen sicherstellen zu können, muss (a) die Bettenmessziffer angehoben, (b) strukturelle Defizite behoben, aber auch (c) innovative Behandlungsmöglichkeiten im Sinne der stationsäquivalenten Behandlung (Hometreatment) umgesetzt werden.

## Abstract (en)

**Background and research question:**

This article presents and analyses the landscape for inpatient child and adolescent psychiatric care in Austria and examines current data from June 2022.

**Method:**

In June 2022, the authors surveyed the current status of child and adolescent psychiatric primary care in Austria. Both systemised beds and day hospital beds as well as the number of specialists and assistant doctors were taken into account.

**Results:**

A bed index of 0.05 beds/1000 inhabitants (as of 6/2022) was calculated, which is below the guideline for beds needed and does not correspond with the need depicted for sufficient provision of care (“Österreichischer Strukturplan Gesundheit”). Moreover, in June 2022, 40 beds had to be closed due to staff shortages and 26.5 training positions were vacant across Austria.

**Discussion and conclusions:**

Even though the training ratio was raised to 1:2 as of 07.02.2022 within the framework of the “Mangelfachverordnung” for a limited period of time until 31.05.2027, it is hardly possible to guarantee sufficient mental health care according to the increased demand for child and adolescent psychiatry, especially in times of the pandemic. Measures need to be urgently taken to improve child and adolescent psychiatric treatment options. In order to ensure adequate care for the increasing number of mentally ill children and adolescents (a) the number of beds must be increased, (b) structural deficits have to improve, and (c) innovative treatment options in the sense of inpatient-equivalent treatment (home treatment) has to be considered.

## Einleitung

Im Rahmen der Arbeitsgemeinschaft Versorgung der Österreichischen Gesellschaft für Kinder- und Jugendpsychiatrie, Psychosomatik und Psychotherapie (ÖGKJP) soll die kinder- und jugendpsychiatrische Versorgungslandschaft in Österreich auf mehreren Ebenen untersucht, ausgewertet und analysiert werden. Dies ist umso bedeutender, da uns die Auswirkungen der Pandemie auf die psychische Gesundheit vor eine große Herausforderung stellen.

Schon in Vergangenheit hat die ÖGKJP Berichte zur Versorgungslage verfasst. Die Autor:innen Fliedl, Ecker und Karwautz [10] untersuchten die fachärztliche Situation und kamen bereits vor der Pandemie zum Schluss, dass zum einen eine große Heterogenität der Versorgungslage in den Bundesländern besteht und zum anderen die Mangelfachregelung nicht ausreichend ist, um die Ausbildung zu konsolidieren und eine Vollversorgung zu erreichen. So sei die vor der Pandemie vereinbarte stationäre Bettenmessziffer (BMZ) von 0,11 in Vorarlberg und Salzburg nahezu erreicht, in der Steiermark und Wien aber mit 0,04 bzw. 0,05 unter der Hälfte des zu erreichenden Wertes. Die Autor:innen gaben an, dass insgesamt österreichweit bei einer BMZ von 0,11 890 voll- bzw. tagesstationäre Plätze zur Verfügung stehen müssten, wovon real im Jahr 2019 lediglich 520 Behandlungsplätze umgesetzt waren.

Inzwischen konnte durch die ÖGKJP erreicht werden, dass mit 07.02.2022 der Ausbildungsschlüssel im Rahmen der Mangelfachverordnung befristet bis 31.05.2027 auf 1:2 angehoben wurde [6].

### Definition der Versorgungsebenen unter Berücksichtigung der gesetzlichen Regelungen

Das Fach Kinder- und Jugendpsychiatrie und -psychotherapeutische Medizin ist im Österreichischen Strukturplan Gesundheit (ÖSG) [7] verankert mit dem Ziel eines flächendeckenden Aufbaus von öffentlichen Versorgungsstrukturen für psychisch kranke Kinder und Jugendliche.

Die Grundlage der integrativen Versorgungsplanung ist die zwischen dem Bund und allen Bundesländern abgeschlossene Vereinbarung gemäß Artikel 15a B‑VG über die Organisation und Finanzierung des Gesundheitswesens sowie das Bundesgesetz zur partnerschaftlichen Zielsteuerung-Gesundheit [5] mit der Festlegung des ÖSG sowie der Regionalen Strukturpläne Gesundheit (RSG) als zentrale Planungsinstrumente.

**Figure 1:**
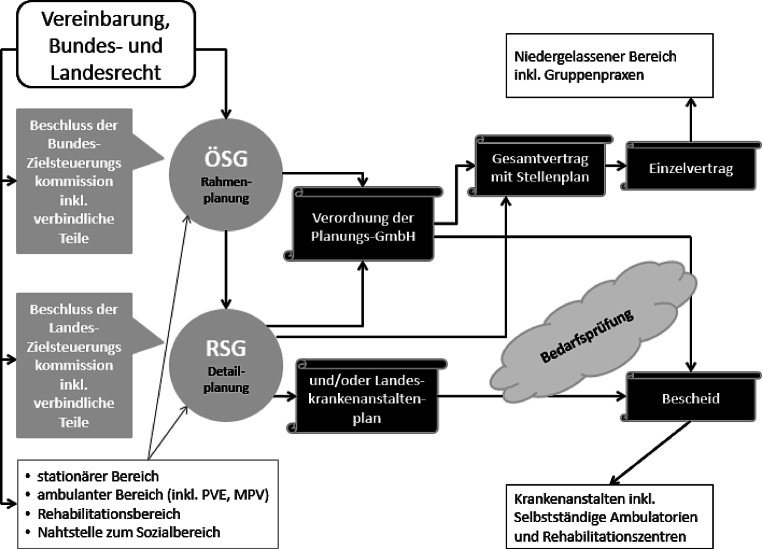

Der ÖSG [7] ist der österreichweit verbindliche Rahmenplan für die in den RSG vorzunehmende konkrete Gesundheitsstrukturplanung und Leistungsangebotsplanung. Er bindet diesbezüglich Bund, Länder und Sozialversicherungsträger (siehe Abb. 1).

Damit legt der ÖSG [7] einen Richtwert für die Ärzt:innendichte im Fachgebiet für jede der 32 Versorgungsregionen in Österreich fest. Allerdings ist die Treffgenauigkeit dieser Richtwerte äußerst gering, da sie zum einen regional nicht ausreichend differenzieren, eine große Bandbreite innerhalb der Versorgungsregionen aufweisen, nicht auf definierten Versorgungszielen basieren und natürlich nicht die aktuellen Veränderungen durch die Pandemie berücksichtigen konnten.

### Definition des Versorgungsauftrages/der Versorgungsprinzipien

Zur Unterstützung der spezifisch notwendigen regionalen Ausgestaltung von Versorgungsangeboten bzw. zur Berücksichtigung regionaler Besonderheiten werden Bandbreiten (Ober- und Untergrenzen) für Planungsrichtwerte angegeben. Ziel ist es, durch das Festlegen von Obergrenzen negative Auswirkungen von allfälliger Angebotsinduktion zu vermeiden sowie durch Untergrenzen Minimalanforderungen an das Leistungsspektrum bzw. an den Versorgungsauftrag in Art und Umfang festzulegen. Die Einhaltung dieser Grenzen ist in der Leistungssteuerung und/oder im Rahmen von regionalen Detailplanungen (RSG) zu beachten. Generell beruhen die Planungsrichtwerte für die Angebotsplanung auf folgenden Annahmen in Bezug auf komplementäre Versorgungsbereiche:

- Sicherstellung einer präklinischen Notfallversorgung durch ein verlässliches Notfallversorgungssystem unter zentraler Einbindung des Notarzt‑/Rettungswesens.
- Sicherstellung einer bedarfsgerechten Verfügbarkeit von Betreuungsangeboten (Rehabilitation, Sozial- und Pflegebereich, Hauskrankenpflege, therapeutisches Angebot) und Unterstützen von Laienhilfe, Selbsthilfegruppen, etc.

Die Grundsätze und Ziele für unser Fach sind der Auf- und Ausbau von stationär und ambulant verschränkten, vorrangig multiprofessionellen Angeboten in der Kinder- und Jugendpsychiatrie (KJP) und Psychosomatik für Kinder und Jugendliche und deren Vernetzung insbesondere mit Angeboten im Sozial- und Bildungsbereich [7].

### Ist-Stand

**Figure 2:**
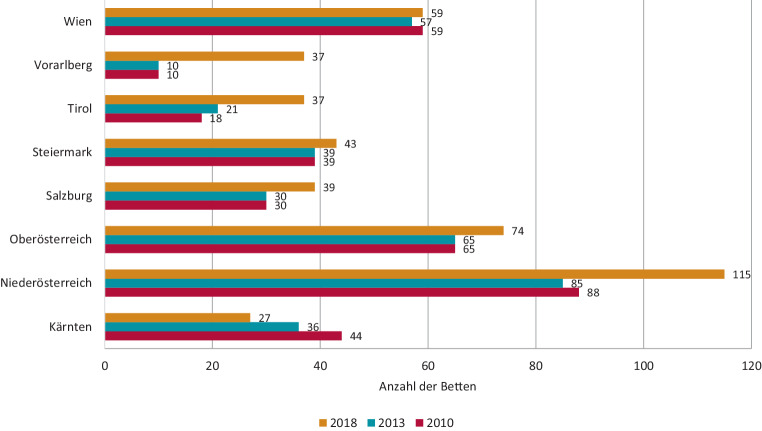

Von der Gesundheit Österreich GmbH (GÖG) wurden die vorhandenen Bettenzahlen im Fach Kinder- und Jugendpsychiatrie in den Jahren 2010, 2013 und 2018 erhoben (siehe Abb. 2). In diesem Zeitraum kamen demnach rund 100 Betten in ganz Österreich hinzu.

Dennoch ist in Österreich keine hinreichende Bettendichte für Kinder und Jugendliche mit psychischen Erkrankungen entsprechend den – vor der Pandemie – definierten Mindestrichtwerten in den einzelnen Bundesländern, mit der Ausnahme von Vorarlberg, gegeben. So hat z. B. das Burgenland keine eigenen stationären KJP-Kapazitäten, stattdessen werden die Kinder und Jugendlichen aus dem Burgenland in Niederösterreich und in der Steiermark mitversorgt, was für die jeweiligen Patient:innen und deren Familien lange Anfahrtswege bedeutet. In den Bundesländern Vorarlberg, Salzburg und Niederösterreich ist die Versorgungsdichte am höchsten [1].

### Bettenmessziffer – Wieviel soll stationär angeboten werden?

In den ÖSG-Verhandlungen hat man sich auf die Zielgröße *Bettenmessziffer* (BMZ, Formel: BMZ = tatsächliche Betten/Gesamtbevölkerung × 1000) geeinigt. Die neun Landesfonds melden jährlich ihre Bettenzahl inklusive ihrer tagesklinischen Behandlungsplätze an die GÖG.

Die BMZ 2018 bezieht sich auf alle für die stationäre Versorgung im Jahr 2018 systemisierten Betten (inklusive Tagklinikplätze). Das Intervall der *Bettenmessziffer vollstationär* (BMZvs) ist Ausdruck der zum Planungshorizont erforderlichen Kapazitätsdichte für die vollstationäre Versorgung der Wohnbevölkerung pro Bundesland. Das Intervall ermöglicht, epidemiologische und intersektorale Versorgungs-Spezifika einer Region zu berücksichtigen und trägt dem heterogenen Ausmaß erzielter Tagklinikanteile bzw. der Verlagerung von Teilen der stationären Versorgung in den spitalambulanten Bereich Rechnung. Die Zurechnung von ausländischen Gastpatient:innen erfolgt zielbezogen (zum Leistungsstandort) gemäß Inanspruchnahme 2018.

Im Rahmen der regionsspezifischen Detailplanung sind Patient:innenströme zu berücksichtigen. Planungsrichtwerte zur Kapazitätsdichte für bettenführende, tagklinische und tagesambulante Strukturen berücksichtigen die regionale Bevölkerungsstruktur und Besiedelungsdichte, die Erreichbarkeitsverhältnisse im Straßen-Individualverkehr (ohne Berücksichtigung wetter-/verkehrsbedingter Verzögerungen), die beobachtete Auslastung bereits bestehender stationärer Einheiten sowie die expertengestützt erwartbaren Tendenzen in der medizinischen Entwicklung in den einzelnen Fach- bzw. Versorgungsbereichen (inklusive der mit dem medizinischen Fortschritt sich ergebenen Möglichkeiten für eine verstärkte Verlagerung in die tagklinische bzw. ambulante Leistungserbringung).

Die GÖG gibt zur Darstellung der erforderlichen Versorgungsstrukturen für das gesamte Leistungsgeschehen in unserem Fach als Planungsrichtwerte drei Kennzahlen mit Bezug auf jeweils 1000 Einwohner:innen an:

- Bettenmessziffer vollstationär für KJP-Plätze
- Bettenmessziffer vollstationär für psychosomatische Plätze
- Bettenmessziffer für Tagklinische Plätze (TK)

Bei den tagklinischen Plätzen ist zu beachten, dass im System der leistungsorientierten Krankenanstaltenfinanzierung (LKF-Modell) 2018 die tagklinischen Plätze im stationären Bettenstand inkludiert waren. Mit Umsetzung der Verlagerung der tagklinischen Behandlungen vom stationären in den ambulanten Bereich werden die tagklinischen Plätze nun mehr in der Kostenstellenstatistik der ambulanten Behandlungsplätze ausgewiesen [7].

### Verweildauer

Nach Angaben der GÖG [7] hat sich auch die Verweildauer inzwischen deutlich reduziert (im Durchschnitt 13,8 Tage bei Buben und 16,6 Tage bei Mädchen), u. a. aufgrund des vorliegenden Aufnahmedrucks bei einer geringen Bettenanzahl, sodass rasch entlassen werden muss und nur wenige Patient:innen längere Therapieaufenthalte erhalten. Dadurch kommt es zu einer relativ hohen Wiederaufnahmerate von ca. 1,7 (siehe Tab. 1).

**Table 1:** 

	Pat	Aufenthalte	Belagstage	Belagstage pro Pat	Belagstage pro Aufenthalt	Wiederaufnahmerate
Männlich	1473	2230	30.741	*20,9*	13,8	1,5
Weiblich	2392	4299	71.488	*29,9*	16,6	1,8
Gesamt	3865	6529	102.229	*26,4*	*15,7*	*1,7*

### Anstieg der psychischen Störungen durch die Pandemie

Inzwischen existieren in vielen westeuropäischen Ländern und auch im deutschsprachigen Raum Erhebungen, welche die psychische Belastung durch die Covid-19 Pandemie bei Kindern und Jugendlichen nachweislich aufzeigen. In der in Deutschland durchgeführten bundesweiten COPSY-Studie (COrona und PSYche) wurde von Mai bis Juni 2020, von September bis Oktober 2021 und im Februar 2022 eine umfangreiche Online-Befragung zur psychischen Gesundheit von Kindern und Jugendlichen und ihren Familien durchgeführt [14]. Es zeigte sich zu allen Befragungszeitpunkten, dass inzwischen fast jedes dritte Kind (vor der Pandemie jedes 5. Kind) psychische Auffälligkeiten zeigt. Während sich bei der ersten Befragung noch keine signifikante Verschlechterung im Bereich der manifesten psychischen Störungen zeigte – es wurde lediglich eine höhere Belastung angegeben – lag zum Zeitpunkt der zweiten und dritten Befragung eine Zunahme an behandlungsbedürftigen psychischen Störungen, vor allem Angst- und depressive Störungen, vor. Die Kinder zeigten zudem häufiger psychosomatische Beschwerden wie Niedergeschlagenheit oder Kopf- und Bauchschmerzen. Etwa 70 % der befragten Kinder und Jugendlichen gaben an, dass sich ihre Lebensqualität durch die Beschränkungen der Covid-19 Pandemie verschlechtert habe. Auch wenn die Werte bei der dritten Befragung sich leicht verbessert haben, blieb eine deutliche Auffälligkeit bestehen. Pieh et al. [13] befragten im Februar 2021 online 3052 österreichische Jugendliche ab 14 Jahren: 55 % wiesen klinisch relevante depressive Symptome auf, 47 % Angstsymptome, 23 % Schlaflosigkeit, 60 % Essstörungssymptome und 37 % Suizidgedanken. In der Tiroler COVID-19 Kinderstudie gaben die im Dezember 2021 befragten Eltern für ihre Kinder (Kindergarten und Grundschule) mehr internalisierende Probleme und posttraumatische Symptome an als die befragten Eltern zu Beginn der Pandemie, das heißt im März 2020 [20].

Die psychische Belastung der Kinder und Jugendlichen zeigte sich auch in den Aufnahmezahlen der kinder- und jugendpsychiatrischen Notaufnahmen. An der Kinder- und Jugendpsychiatrie der Universität Tübingen zeigte sich während des zweiten Lockdowns ein Anstieg der Notfälle um 30 % [15]. An der Abteilung für Kinder- und Jugendpsychiatrie Hall i. T./Innsbruck stiegen die Akutaufnahmen im zweiten Pandemiejahr 2021 um 40,1 % im Vergleich zum Vor-Corona-Jahr 2019. In der Covid-19 Pandemie nahm die akute Suizidalität um 48,3 % zu, wohingegen die Fremdaggression um −51,0 % abnahm [16].

## Aktuelle Bettenmessziffern

### Tatsächliche Bettenmessziffern laut Österreichischem Strukturplan Gesundheit Monitoring 2021

Die in Tab. 2 angeführten Zahlen zur BMZ (Soll) sind Vorgaben des ÖSG [7]. Da in den meisten Abteilungen für KJP sowohl kinder- und jugendpsychiatrische als auch psychosomatische Plätze vorhanden sind, wurden diese beiden Planungsrichtwerte für den gesamten kinder- und jugendpsychiatrischen stationären Bereich zum Zweck der Vergleichbarkeit von den Autorinnen zusammengefasst und ergeben eine *BMZ stationär gesamt* von 0,07 bis 0,13.

**Table 2:** 

BMZ: KJP-stationär	0,05 bis 0,09
BMZ: psychosomatisch	0,02 bis 0,04
BMZ: TK	0,04

Aufgrund der Angaben der Krankenkassen-Kostenstellenstatistik 2020 sowie Auskünften der Einrichtungen liegen im ÖSG-Monitoring 2021 [19] Zahlen über die tatsächlichen Betten vor. Auf dieser Basis wurde die entsprechende BMZ errechnet (siehe Tab. 3). Die tatsächliche BMZ für die kinder- und jugendpsychiatrischen Betten liegt mit 0,04 außerhalb des Planungsrichtwertes von 0,05–0,09. Ebenso liegt die tatsächliche BMZ für psychosomatische Betten mit 0,01 außerhalb des Planungsrichtwertes von 0,02–0,04. Die tatsächliche BMZ für tagklinische Plätze liegt mit 0,01 am weitesten außerhalb des Planungsrichtwertes von 0,04.

**Table 3:** 

	Krankenanstalten-Kostenstellenstatistik 2020	Tatsächliche Bettenmessziffer
BMZ: KJP-stationär	349 Betten	0,04
BMZ: Psychosomatisch	120 Betten	0,01
BMZ: TK	116 Plätze	0,01

Fasst man die BMZ für KJP und psychosomatische Betten zusammen, liegt die tatsächliche BMZ mit 0,05 unter dem Planungsrichtwert laut ÖSG von 0,07 bis 0,13 [7].

### Methode: Aktuelle eigene Befragung mit Stand 6/2022

Da die oben angegebene Krankenkassen-Kostenstellenstatistik aus dem Jahr 2021 stammt und die Auswirkungen der Pandemie an einigen Standorten zu Bettensperrungen wegen Personalmangels geführt haben, wurde von den Autorinnen im Rahmen dieses Artikels eine aktuelle Befragung mit Stand 6/2022 durchgeführt und in Bezug zur Einwohner:innenzahl gesetzt (das heißt die BMZ berechnet). Die aktuellen Betten (systemisierte Betten, TK-Plätze, Betten wegen Personalmangel gesperrt) und die personelle ärztliche Ausstattung (Fachärzt:innen (FÄ), Ausbildungsstellen Ärzt:innen KJP, unbesetzte Ausbildungsstellen Ärzt:innen KJP) wurden von den Autorinnen im Juni 2022 schriftlich an allen österreichischen kinder- und jugendpsychiatrischen Primariaten abgefragt. Psychosomatische Betten an der Pädiatrie wurden in der Befragung hingegen nicht berücksichtigt.

### Ergebnisse: Aktuelle eigene Befragung mit Stand 6/2022

In Österreich gibt es (Stand 6/2022) 437 vollstationäre und 138 TK-Plätze (siehe Tab. 4) für 1,64 Mio. Kinder und Jugendliche unter 19 Jahren [17]. Das ergibt eine BMZ von 0,05 für die KJP-stationären inklusive der psychosomatischen Plätze (wobei psychosomatische Betten an der Pädiatrie in der aktuellen Befragung keine Berücksichtigung fanden).

**Table 4:** 

Bundesländer	Versorgungsregionen	Einwohner: innen pro Bundesland^a^ [17]	Einwohner: innen 0–18 J. pro Bundesland^a^ [17]	Systemisierte Betten (KJP & Psychosomatik^b^)	TK-Plätze	Betten wg. Personalmangel gesperrt	FÄ	Ausbildungsstellen Ärzt:innen KJP	Unbesetzte Ausbildungsstellen Ärzt:innen KJP
Burgenland	Burgenland	297.583	50.096	0 (ab 2027: 20)	0	0	6	1	1
Kärnten	Klagenfurt	564.513	96.166	31	8	0	6	7	5
Niederösterreich	Hinterbrühl (NÖ und nördl. Burgenland)	1.698.796	3035	30	16 (ab Okt. 2022: 20)	0	9 (3 VZÄ)	9 (um 12 angesucht mit neuer Regel)	0 (bei Bewilligung 3)
	Mauer bei Amstetten			30	6	0	6 (3 VZÄ)	4	2
	Tulln			20 (ab 2024: +4 Eltern/Kind)	10	0	8	7	1
Oberösterreich	Linz, 2 Standorte	1.505.140	288.195	54	22	0	11	6	3
	Grieskirchen			27	5	0	4	4	4
Salzburg	Salzburg	562.606	104.398	30	10	0	7	7	0
	Schwarzach im Pongau			12	0	0	2	2	0
Steiermark	Graz (Steiermark und südl. Burgenland)	1.252.922	214.059	33 (ab 2023: 53)	14 (ab Okt. 2022: 20)	0	14 (10,6 VZÄ)	10 (7,8 VZÄ)	0
Tirol	Hall i. T.	764.102	140.457	37	6	0	7	10	4
	Innsbruck				5			1	0
Vorarlberg	Bregenz	401.674	81.086	36	8	1	5	5	1
	Rankweil				4				
Wien	Medizinische Universität Wien	1.931.593	350.695	30	8	6	12	13	0
	Wien, Floridsdorf			24	6	24	2,25	0	0
	Wien, Klinik Hietzing			43	0	9	5,5	17	6
	Wien, Psychosoziales Netzwerk			0	10 (–12)	0	2,5	2	1,5
*Österreich*	**–**	*8.978.929*	*1.639.187*	*437*	*138*	*40*	*106,25*	*105*	*26,5*

*NÖ* Niederösterreich, *OÖ* Oberösterreich, *VZÄ* Vollzeitäquivalente

^a^Per 01.01.2022

^b^Ausschl. KJP-psychosomatische Betten, d. h. ohne psychosomatische Betten an der Pädiatrie

## Diskussion

Im vorliegenden Artikel wurde die stationäre kinder- und jugendpsychiatrische Versorgungslandschaft in Österreich vorgestellt und analysiert und aktuelle Daten aus dem Juni 2022 untersucht. Es wurde eine Bettenmessziffer von 0,05 (Stand 6/2022) berechnet, die unter den vom Österreichischen Strukturplan Gesundheit [7] vorgegebenen Planungsrichtwerten liegt. Im Juni 2022 waren österreichweit 40 Betten wegen Personalmangels geschlossen und 26,5 Ausbildungsstellen unbesetzt.

Darüber hinaus ist davon auszugehen, dass durch die zunehmende Verschlechterung der psychischen Gesundheit unserer Kinder und Jugendlichen, unter anderem aufgrund der Covid-19 Pandemie und der damit einhergehenden Eindämmungsmaßnahmen (z. B. [13, 14, 20]), die bisherigen Planungsrichtwerte aus dem Jahre 2017 nicht mehr den realen Bedarf abbilden. Internationale [8, 11], aber auch deutsche [15] und österreichische [16] Studien zeigen einen Anstieg der Akutaufnahmen an der Kinder- und Jugendpsychiatrie während der Covid-19 Pandemie. Abgesehen von den Herausforderungen und Belastungen durch die Pandemie, möchten wir festhalten, dass die Planungsrichtwerte bezüglich Bettenmessziffern im kinder- und jugendpsychiatrischen Bereich, wie dargestellt, auch zuvor nur in den Bundesländern Vorarlberg und Salzburg nahezu erreicht wurden, in anderen Bundesländern hingegen nur zur Hälfte. Dies zeigte sich unter anderem in hohen Aufnahmezahlen und verringerten Verweildauern, da der sich erhöhenden Zahl an Notaufnahmen nur durch verkürzte Aufenthalte noch begegnet werden konnte [7].

Dies allerdings führt schnell zur Gefahr eines „Drehtüreffekts“, da nicht hinreichend behandelte Jugendliche oftmals Gefahr laufen, sich schnell erneut zur Aufnahme vorstellen zu müssen [9]. Auch sollte einbezogen werden, dass sich vor allem internalisierende Erkrankungen wie Ängste und Depressionen zu erhöhen scheinen [4], Erkrankungen die, wenn sie einmal manifest sind, lange Behandlungsverläufe bedingen, da sie langsam schleichend entstehen und dadurch eher dazu tendieren zu chronifizieren [18]. Dem sollte auch in Österreich begegnet werden. Dabei wäre es wünschenswert, nicht nur eine dringend nötige Erhöhung der BMZ und somit auch des Personalschlüssels zu erwirken, sondern auch innovative Strategien wie Hometreatment im LKF-Schlüssel zu verankern, so dass Familien dort Hilfe erfahren, wo diese am dringendsten nötig ist, nämlich zu Hause vor Ort.

Gleichzeitig müssen – um über die KJP-Versorgung regelmäßig berichten, bzw. diese evaluieren zu können – maßgebliche Bedarfskennzahlen erfasst und ausgewertet werden. Nur so kann eine gesetzlich vorgegebene ausreichende Versorgung quantitativ und qualitativ beurteilt werden. Ausstattungsmerkmale wie Vorhalten von Fachtherapien und ein Mindestmaß an umzusetzenden Behandlungsstunden als auch Behandlungsstunden pro Ärzt:in bzw. Psycholog:in/Patient:in sollten festgehalten werden, um vergleichbare Kennzahlen zu erhalten. Dabei muss insbesondere einbezogen werden, dass eine ärztliche Besetzung in der KJP immer noch schwierig ist und die KJP so als Mangelfach gilt. Dies ist sicherlich anteilig auch der Fall, da die Lehre nicht an allen Universitäten hinreichend verankert ist. Es bedarf deswegen eines Österreichweiten, jährlichen standardisierten Monitoring der besetzen und unbesetzten ärztlichen Planstellen verbunden mit geeigneten Maßnahmen zur Sicherstellung der Versorgung der Bevölkerung, z. B. vermehrten ambulanten Angeboten in der Breite, Etablierung von Hometreatment-Teams [3], niederschwelligen Angeboten an Schulen [2], psychosozialen Diensten, die Hand in Hand arbeiten, aber auch einer ausreichenden Zahl an systemisierten Betten, um nicht nur stationäre Notfallbehandlungen, sondern auch nötige Therapieplätze stationär und teilstationär anbieten zu können. Dabei sind nicht nur (Fach‑)Ärzt:innen zur Besetzung der Teams ausschlaggebend, sondern gerade in der KJP spielen Ressourcen der Multiprofessionalität eine große Rolle, so dass unter Supervision und Fallletztverantwortlichkeit einer/eines Fachärzt:in Behandlung auch von anderen Berufsgruppen professionell und für die Kinder, Jugendlichen und Familien hilfreich erfolgen kann.

## Umsetzungsempfehlungen/Schlussfolgerung

Psychische Störungen im Kindes- und Jugendalter werden als sogenannte „neue Morbidität“ angesehen, die mit einer hohen Krankheitslast einhergehen, zu Chronifizierungen neigen, transgenerationale Effekte zeigen und nicht selten mit einer lebenslangen Teilhabebeeinträchtigung einhergehen können [12]. Diese Tatsachen waren bereits vor Ausbruch der Pandemie bekannt, sind nun aber durch die Pandemie noch verstärkt worden.

Daher ist es dringend notwendig, dass Maßnahmen zur Verbesserung der kinder- und jugendpsychiatrischen und -psychotherapeutischen Behandlungsmöglichkeiten ergriffen werden. Es bedarf ein für jeden – gerade die sozial schwachen Familien – erreichbares, abgestuftes Angebot, von ambulant über teilstationär, über Hometreatment bis hin zu stationären spezifischen Behandlungskonzepten, um nicht nur Notfallmanagement zu betreiben, sondern eine angemessene Versorgung der zunehmenden Zahl an manifest psychisch erkrankten Kindern und Jugendlichen sicherstellen zu können.

Die BMZ muss angehoben, strukturelle Defizite behoben, aber auch innovative Behandlungsmöglichkeiten im Sinne der stationsäquivalenten Behandlung (Hometreatment) angedacht werden, um Patient:innen bedarfsgerecht versorgen zu können.

Folgende Punkte sollten hierfür zeitnah umgesetzt werden:

- Verbesserung der strukturellen stationären Defizite durch Anpassung des (**Fach‑)Ärzt:innenschlüssels und der BMZ/Bundesland**. Nur so ist eine leitliniengerechte facharztgesteuerte Behandlung unter Berücksichtigung des zunehmenden Bedarfs an intensiver kinder- und jugendpsychiatrischer Behandlung durch die Pandemie abzubilden.
- Schaffung entsprechend hinreichender Ausbildungsstellen.
- **Durchgängiges Etablieren von multiprofessioneller Behandlung** durch entsprechendes Vorhalten von Kapazitäten in der Kinder- und Jugendhilfe- sowie im pädagogischen/schulischen Bereich (Schulsozialarbeiter:innen, Schulpsycholog:innen, Schulpsychotherapeut:innen, Schulärzt:innen), um Kinder und Jugendliche dort wieder in die Gesellschaft zu integrieren, wo sie durch die Covid-19 Pandemie oftmals isoliert worden sind.
- **Implementierung neuer Versorgungsmodelle** wie das multiprofessionelle Hometreatment als stationsersetzende Maßnahme in den LKF-Schlüssel und Umsetzung von entsprechenden Angeboten, um die Möglichkeit zu haben in den Familien vor Ort zu arbeiten und so das familiäre Umfeld intensiv in die Behandlung mit einbeziehen zu können.
- **Enge Vernetzung mit psychosozialen Strukturen**, um Ressourcen zu bündeln und zeitnahe Übergänge gestalten zu können
- Etablierung eines Ministerien-übergreifenden Koordinators für Mental Health.
